# Pathophysiology of Aortic Aneurysms: Insights from Animal Studies

**DOI:** 10.26502/fccm.92920146

**Published:** 2020-08-31

**Authors:** Mitri K Khoury, Amelia R Stranz, Bo Liu

**Affiliations:** 1 Department of Surgery, Division of Vascular and Endovascular Surgery, University of Texas Southwestern Medical Center, Dallas, United States; 2 Department of Surgery, Division of Vascular Surgery, University of Wisconsin-Madison, WI, United States

**Keywords:** Aortic aneurysm, Treatment, Surgery, Mouse model, Cell biology, Molecular biology, Pathophysiology

## Abstract

Aortic aneurysms are defined as dilations of the aorta greater than 50 percent. Currently, the only effective treatment for aortic aneurysms is surgical repair, which is recommended only to those that meet criteria. There is no available pharmaceutical therapy to slow aneurysm growth and thus prevent lethal rupture. The development of a number of murine models has allowed in depth studies of various cellular and extracellular components of aneurysm pathophysiology.

The identification of key therapeutic targets has resulted in several clinical trials evaluating pharmaceutical candidates to treat aneurysm progression. In this review, we focus on providing recent updates on developments in murine models of aortic aneurysm. In addition, we discuss recent studies of the various cellular and extracellular components of the aorta along with the abutting aortic structures that contribute to aneurysm development and progression.

## 1. Introduction

Aortic aneurysms are defined as dilations greater than 50 percent of the normal aortic diameter. Formation of aneurysms can occur anywhere along the length of the aorta, but infrarenal abdominal aortic aneurysms (AAAs) are the most common. The annual incidence of a new AAA is approximately 0.4 to 0.7 percent, predominantly affecting men older than 65 [1]. A feared complication of aneurysms is rupture, which has an associated mortality rate of 80 percent [2]. Therefore, preventing aneurysm rupture has been the primary goal of AAA management. Current treatment strategies include open and endovascular repair of aneurysms. Surgical interventions are effective in preventing aneurysm rupture; however, are only recommended to AAAs that meet size criteria (>5.5cm for men and >5.0cm for women), expand rapidly, or are symptomatic. Aneurysms that do not meet these criteria are typically surveilled via ultrasound or computed topography (CT). The unmet clinical need for pharmaceutical agents capable of attenuating aneurysm growth and rupture has motivated a wide range of clinical and basic investigations. Another area of active research is aneurysm etiology. Several risk factors for developing an aneurysm have been identified. Tobacco use is a modifiable risk factor that is most strongly associated with aneurysm development [3]. Studies analyzing human aortic tissue showed that patients who smoke have significant reductions in the elastin matrix within the aortic wall [4]. These patients have increased levels of matrix degrading proteases, inflammatory cells, and evidence of mitochondrial stress within the aortic wall, as well as dysfunctional repair mechanisms that ultimately lead to aneurysm formation [5]. Other potentially modifiable risk factors for aortic aneurysms are hypertension [6] and atherosclerosis [7]. Non-modifiable risk factors include familial predisposition [8] and male sex [9]. Thoracic aortic aneurysms are frequently found in patients with rare genetic diseases, such as Marfan syndrome (fibrillin-1 defect), Ehlers-Danlos syndrome, and Loeys-Dietz syndrome. However, a positive family history alone is a significant risk factor for developing aneurysms independent of the aforementioned syndromes [10].

## 2. Murine Models of Aortic Aneurysms

Mouse models of aortic aneurysms are instrumental to studying aortic aneurysm and to identify potential therapeutic targets for this disease. Many of the models recapitulate the major characteristics of human aortic aneurysm formation, such as medial degeneration, vascular smooth muscle cell (VSMC) death, and inflammation. These models, particularly when used in conjunction with gene knockout, gene editing, or transgenic technologies, have contributed significantly to our understanding of the pathophysiology of aortic aneurysm. Creation of aortic aneurysms in mice can be categorized by three major methods: genetic, chemical, or hybrid of both approaches. Genetic approaches consist of creating genetic or spontaneous mutations, whereas, chemical approaches include intraluminal perfusion, periaortic application, or subcutaneous infusion of various molecules.

### 2.1 Genetic approaches to aortic aneurysm induction

Several genetically modified mice develop spontaneous aortic aneurysms, including blotchy (mutation on the X chromosome); *Lox*^*−/− mice*^, and mice with deficiencies in metalloproteinases or their inhibitors (*Mmp3*^*−/−*^ and *Timp1*^*−/−*^). The blotchy and *Lox*^*−/−*^ mice are rarely used as they have inherent problems with viability and significant vascular pathology outside of the aorta [11, 12]. The *Mmp3*^*−/−*^ and *Timp1*^*−/−*^ mice develop small spontaneous dissecting aneurysms along the entire length of the aorta as well as in other segments of the arterial system [13, 14]. Thus, this model has been criticized for reflecting systemic arterial extracellular matrix destruction and lacking specificity to aortic aneurysms. Marfan syndrome is an autosomal dominant connective tissue disease that results from mutations in *Fbn1* encoding the protein fibrillin 1. Fibrillin 1 is critical in maintaining the integrity of the extracellular matrix. As a result, patients with Marfan syndrome often experience aneurysms and/or dissections of their aorta, specifically the thoracic segment. Several mouse models have been generated to mimic this disease process via homozygous mutations in the fibrillin-1 gene [15]. Similar to humans, mice with fibrillin-1 mutations develop aneurysms at or near the aortic root and die from either rupture or dissection. Thus, this disease model has been useful in studying mechanisms and effectiveness of various therapeutic strategies in Marfan syndrome.

### 2.2 Angiotensin II (Ang II) model

One of the most commonly used mouse models of aortic aneurysm is the Angiotensin II (Ang II) model [16]. This model typically consists of utilizing either *Ldlr*^*−/−*^ or *Apoe*^*−/−*^ mice and subjecting them to Ang II infusion via a subcutaneous pump [17–19]. Aorta are typically harvested 14–28 days after pump insertion and are localized to the thoracic and suprarenal abdominal aorta. Further, this model generally creates dissecting aneurysms and have a high mortality rate due to rupture. While the purpose of utilizing the *Ldlr*^*−/−*^ or *Apoe*^*−/−*^ mice is to generate hypercholesterolemia, other variations of this model have allowed investigators to use mice of different backgrounds. Recently, Lu et al. reported successful induction of aortic aneurysm via Ang II in C57BL/6 mice with hypercholesterolemia that was induced by an adeno-associated viral vector expressing a gain-of-function mutation of PSCK9 [20]. This modified approach provides an alternative method of generating hypercholesterolemia in knockouts of interest without crossbreeding with *Ldlr*^*−/−*^ or *Apoe*^*−/−*^mice. Experimentally, degeneration of the elastic lamina can be induced via administration of ß-aminopropionitrile (BAPN). BAPN is an inhibitor of lysyl oxidase, which plays a critical role in cross-linking elastin and collagen fibers [21]. Lysyl oxidase activity significantly decreases with age [22]. and; therefore, administration of BAPN is thought to mimic human aging [23]. Kanematsu et al. used a combination of Ang II and BAPN infusion to induce aortic aneurysms in C57BL/6J mice and found that both thoracic and abdominal aortic aneurysms were created [24]. Interestingly, they found that amlodipine, but not captopril, was able to reduce both hypertension and aneurysm formation in mice under this model. Thus, the authors concluded that aneurysm formation in this model was dependent on hypertension and not on the direct effects of Ang II on the aortic wall. Lastly, a unique benefit of this version of the Ang II model is that it does not require a hypercholesterolemic state. Most chemically induced aneurysm models, including the Ang II model, are criticized for its acuity when, in reality, human aortic aneurysms are chronic in nature. To this end, Rateri et al. created a two-pump model to mimic a chronic disease [25]. Male *Apoe*^*−/−*^ mice were subjected to Ang II pump infusion for 27 days and then either subjected to a second Ang II infusion or to saline for additional 28 days. Rateri et al. found that mice subjected to saline infusion did not experience any further dilatation or aneurysm-related deaths. In contrast, mice subjected to a second pump of AngII infusion had continued aneurysmal dilation, ruptures, and aortic remodeling. Thus, the continued Ang II infusion model provides investigators a way to study chronic aneurysmal changes. However, a significant drawback of this method is the length of time required to complete *in vivo* studies.

### 2.3 Calcium chloride

Perivascular application of calcium chloride was first conducted by Gertz et al. who applied 0.5M CaCl_2_ to the commom carotid artery of rabbits, which led to aneurysm formation [26]. Subsequently, CaCl_2_ was applied to the abdominal aorta in rabbits [27] and then rodents [28] to induce AAAs. T+o induce AAA formation. The application of periaortic CaCl_2_ results in various cellular effects within the aortic wall that mimic human aortic aneurysms. A large inflammatory reaction occurs with infiltration of neutrophils, macrophages, and monocytes which is accompanied by changes in cytokine and proteinase profiles [29]. Further, the CaCl_2_ model leads to VSMC death and extracellular matrix remodeling [30]. Traditionally, this model consists of applying 0.5M CaCl_2_ soaked gauze around the infrarenal abdominal aorta for 10 minutes. Our lab expanded this model by introducing an additional application of phosphate buffer following CaCl_2_ application [31]. We found that when mouse aortae are treated with 0.5M CaCl_2_ for 10 minutes, followed by phosphate-buffered saline for 5 minutes, they developed a more robust aneurysm compared with those treated with CaCl_2_ alone.

### 2.4 Elastase

Infusion of porcine pancreatic elastase via a catheter within the lumen of the infrarenal segment of the aorta was first introduced in rats and subsequently adapted to mice by Thompson and colleagues [32]. In this robust and reproducible model, aneurysms usually develop by day 14 and are accompanied by elastin degradation and inflammatory infiltrate [33]. However, this model can be very technically challenging. Bhamidipati et al. generated a variation of this model in which they subjected mice to periadventitial application of porcine pancreatic elastase [34]. Aneurysms were generated in 82% of mice and were accompanied by elastin degradation, macrophage infiltration, and matrix metalloproteinase-9 expression. Thus, periadventitial application of porcine pancreatic elastase appears to be a good alternative that avoids the technical challenges associated with the intraluminal approach. However, the elastase model has been scrutinized due to its acuity, lack of sustained aneurysmal dilation, and inability for transmural rupture of the aortic wall, which are characteristics of human aneurysms. Therefore, modifications of the elastase model have been sought in order to better mimic human aneurysms. TGFβ activity is well-known for its importance in maintaining aortic wall integrity. Studies have shown that disruption of TGFβ signaling leads to substantial increase vascular wall inflammation and ECM degradation [35–36]. Therefore, Lareyre et al. conducted a periadventitial application of elastase on the aortic wall of mice along with injections of mouse anti-mouse TGFβ [37]. They found that this model led to sustained aneurysmal growth, the development of intraluminal thrombus, and aortic wall rupture. Further, TGFβ blockade enhanced leukocyte infiltration both in the aortic wall and intraluminal thrombus, which is a classical characteristic of human aortic aneurysms. Another modification addressing the limitations of the original elastase model was created by Lu et al. utilizing the Lox inhibitor BAPN [38]. BAPN was added to the water of mice 2 days prior to periaortic application of the aorta until the end of the study. Mice given BAPN demonstrated continued long-term growth, thrombus formation, and aortic rupture for as long as 100 days. Thus, these modifications of the elastase model are thought to closely resemble the chronic degenerative nature seen in human aneurysms.

## 3. Cellular Responses of the Aortic Wall

The primary cellular components of the aortic wall consist of endothelial cells, smooth muscle cells, and fibroblasts. These cells are organized in three layers: intima, media, and adventitia. The vessel wall also contains inflammatory cells such as residential macrophages. Additional inflammatory cells can be recruited from the circulation during vascular injury. Human aneurysmal tissues exhibit substantial abnormalities that affect cellular components and the extracellular matrix of the aortic wall. Experimental evidence obtained from animal studies suggests that the complex changes in primary vascular cells, extracellular matrix, and inflammatory cells are critical to the development of an aortic aneurysm. Below we discuss the major cellular components and their contribution to the pathogenesis of aortic aneurysms.

### 3.1 Inflammatory cells

It is well established that human aortic aneurysms contain leukocytes of multiple populations [39]. Localized structural deterioration is frequently accompanied by accumulation of macrophages within aneurysmal tissues. Targeting the inflammatory process has been a major focus of therapeutic development aiming to halt aneurysm progression. Umebayashi et al. highlighted the therapeutic benefit of reducing inflammation in the aortic wall by treating mice with cilostazol, a phosphodiesterase III inhibitor commonly used in patients with peripheral vascular disease [40]. They found that cilostazol treatment attenuated aortic aneurysm progression via reduction in expression of inflammatory cytokines within the endothelium, which subsequently led to a significant decrease in macrophage accumulation as well as matrix metalloproteinase activities. It is generally believed that macrophages exist in a spectrum of functional phenotypes [41]. The two polarizing ends are the proinflammatory (M1) and anti-inflammatory (M2) macrophages. M1 macrophages are traditionally thought to be the driving force of aneurysm formation, whereas M2 macrophages are thought to be protective of aneurysms. This concept was supported by Batra et al. who demonstrated in a mouse model of AAA that TNFα-deletion inhibited M1 macrophage polarization and aneurysm formation [42]. Further, infusion of M1 polarized *TNFα*^*−/−*^ macrophages inhibited growth of aortic aneurysms. However, a study by Sharma et al. using *Il12p40*^*−/−*^ mice countered the traditional concept that M2 macrophages are beneficial in aortic aneurysm formation [43]. Interleukin 12 (Il12), a heterodimeric cytokine composed of p35 and p40 subunits, has been shown to be a key regulator of macrophage polarization. Previous studies have reported that macrophages deficient in IL12p40 are biased towards an M2 profile [44]. Sharma et al. found that IL12p40 depletion promoted the development of abdominal aortic aneurysm by facilitating the recruitment of M2-like macrophages. Thus, the concept of M2 macrophages as beneficial in aneurysms may not be as clear as once thought.

Other leukocyte populations including T-cells and neutrophils have been implicated in the pathophysiology of aortic aneurysms [45]. CD4^+^CD25^+^Foxp^+^ regulatory T cells (Tregs) account for a minority of the total T cells, but have important functions in regulating autoimmunity [46]. Previous reports have found that Treg depletion significantly increased aortic aneurysm development [47–48]. Similarly, Li et al. found that exogenous treatment of interleukin 33 (IL-33) reduced aneurysm progression and aortic wall inflammation [49]. Treatment with IL-33 increased Tregs within the aorta and suppressed vascular smooth muscle cell (VSMC) chemokine expression. Suh et al. also explored the role of Tregs in aneurysm development by using a humanized murine model of AAA by irradiating *Rag1*^*−/−*^ mice at 7 weeks of age and supplementing them with human CD4^+^ T-cells [50]. *Rag1*^*−/−*^ mice are resistant to aneurysm formation in the CaCl_2_ model; however, when supplemented with human CD4^+^ cells, they became susceptible to AAA formation. Furthermore, augmentation of human Tregs via interleukin-2 led to decreased aneurysm progression in this model. This study suggests that expansion of Tregs may be a potential therapeutic target in AAAs. In a study by He et al., the investigators explored the role of FAM3D (family with sequence similarity 3, member D), a recently identified chemokine, in neutrophil recruitment and aneurysm development [51]. He and colleagues found that FAM3D induced Mac-1-mediated neutrophil recruitment and aggravated aneurysm formation through formyl peptide receptor-related Gi protein and β-arrestin signaling. This finding highlighted the importance of the chemokine-leukocyte interaction in the development of aortic aneurysms. IL-1β has long been known to play an important role in promoting the inflammatory processes of various diseases. As such, IL-1β has been an intriguing therapeutic target for various diseases [52]. Johnston et al. found that IL-1β mRNA and protein levels were significantly higher in aortas of mice that underwent elastase perfusion [53]. Similarly, genetic deletion of IL-1β or IL-1R demonstrated protection against AAA formation in the elastase model. However, this contradicts Batra et al. who found that genetic deletion of IL-1β or IL-1R did not block aneurysm formation in the CaCl_2_ model [42]. Batra and colleagues postulated that the lack of effects was primarily because of a skewing to M1 macrophages secondary to IL-1β deficiency. Consistently, a clinical trial evaluating the effect of monthly subcutaneous injections of canakinumab, an IL-1β inhibitor, was terminated due to its lack of efficacy in treating aortic aneurysms (NCT02007252).

### 3.2 Extracellular matrix remodeling

The extracellular matrix (ECM) is the acellular component of the aorta that provides structural support and regulates the bioavailability of various cytokines and growth factors. Loss of proper ECM structure and homeostatic maintenance is thought to be a significant contributor to the pathophysiology of aortic aneurysms [54]. Inflammation and remodeling of the extracellular matrix are thought to be closely linked, which leads to profound inflammatory responses, matrix degeneration, and elastin fragmentation. Therefore, major research efforts have been devoted to understanding ECM remodeling during aneurysm development. Interleukin-10 (IL-10) is a well-known cytokine with anti-inflammatory properties. Mice deficient in IL-10 develop larger aortic aneurysms, raising the possibility that its administration may be of therapeutic benefit [55]. Adam et al. subsequently used IL-10 minicircle transfection into *ApoE*^*−/−*^ mice to increase bioavailability [56]. Minicircles are episomal DNA vectors that are used for genetic modification of mammalian cells. Minicircles typically have higher transfection and survival rates in target cells compared to plasmids [57–58]. Using this method, Adam and colleagues demonstrated a significant reduction in aortic diameter, markers of inflammation, and improved aortic remodeling in the Ang II model [56]. The study highlighted not only that IL-10 can be a potential therapeutic option to improve aortic option, but minicircles may be an intriguing vector for gene therapy. Yin et al. found that glycosylation of microfibril-associated glycoprotein 4 (MFAP4) significantly differed in patients with Marfan syndrome [59]. Further, these ECM changes were accompanied by an abundance of proteases from the ADAMTS (a disintegrin-like and metalloprotease domain with thrombospondin-type motifs) family. The ADAMTS family of proteases are the main enzymes responsible for cleaving large aggregating proteoglycans. Interestingly, both Fava et al. and Dupuis et al. recently demonstrated the importance of ADAMTS-5 in maintaining ECM homeostasis via its action on various proteoglycans [60–61]. Fava and colleagues demonstrated regional differences in ADAMTS expression in murine aortas and showed that ADAMTS-5, not ADAMTS-1, was the key protease for versican regulation in thoracic aortic aneurysms. Similarly, Dupuis and colleagues found that disruption of ADAMTS-5 cleavage during development was associated with ascending aortic anomalies. Together, data from these two studies suggest that while dysregulation of the ADAMTS family leads to aortic aneurysms via disruption of the ECM, ADAMTS-5 may be specific to the thoracic region.

Clinically, as well as experimentally, aortic dissections may undergo remodeling that subsequently leads to aneurysms. Angiotensin converting enzyme inhibitors are found to prevent cardiac remodeling after myocardial infarctions; for this reason, the drugs are often prescribed in patients with coronary artery disease [62]. The renin-angiotensin signaling system has an established role in the development of aortic aneurysms [63]. Ang II has been shown to play a role in aortic aneurysms in mice and functions through two main receptors: Ang II Type 1 receptor (AT1R) and Ang II type 2 receptor (AT2R). In the context of aortic aneurysm, AT1R is likely to be the predominant receptor responsible for disease formation [64]. In contrast, AT2R appears to have a protective role in aortic aneurysm formation [65]. However, a recent study tested the effect of compound 21 (C21), a selective nonpeptide AT2R agonist, on aortic root enlargement in a Marfan mouse model (*Fbn1*^*C1039G/+*^) and found no effect on aneurysm growth or remodeling [66]. Zhou et al. used a transverse aortic constriction (TAC) model to further elucidate the role of AT2R and MasR on aortic remodeling by using various combinations of losartan (AT1R inhibitor), captopril (ACE inhibitor), C21, and PD123319 (AT2R inhibitor) [67]. They found that there was a beneficial effect of AT1R blockage following TAC-induced aortic remodeling, which is dependent on AT2R activation. This finding suggests that ACE inhibitors may not be as effective as losartan in preventing aortic remodeling.

### 3.3 Vascular smooth muscle cells

Medial VSMC death is integral to the development of aortic aneurysm formation as depletion of VSMC is a major pathological characteristic of human aortic aneurysm. Traditionally, both apoptosis and necrosis were thought to be essential for VSMC death [68–69]. However, as knowledge of cell death expands, other forms of cell death are found to play a role in VSMC depletion. Necroptosis is a form of regulated necrosis that is mediated by receptor interacting protein kinase 3 (RIPK3). Levels of RIPK3, along with its partner in death RIPK1, were found to be elevated in human aortic aneurysm samples [70]. Experimental data from our lab showed that deletion of *Ripk3* prevented AAA formation by protecting against loss of VSMCs and by impairing inflammatory gene expression. Further, we demonstrated, in principle, that inhibition of this necroptotic pathway may attenuate aneurysm formation and block disease progression using small chemical inhibitors to RIPK1 and RIPK3 [70–73]. Emerging evidence suggests that VSMCs contribute to aneurysm pathophysiology through mechanisms beyond cell death. VSMCs express unique contractile proteins, ion channels, and signaling proteins that are essential to proper contraction and relaxation of blood vessels [74]. Contrary to skeletal and cardiac myocytes, VSMCs have remarkable plasticity and can undergo a variety of phenotypic changes including loss of contractile proteins [75]. These phenotypic changes are typically a response from signaling molecules in the local environment. Bogunovic et al. studied the *in vitro* contractility via electric cell-substrate impedance sensing (ECIS) of biopsied healthy human and aneurysmal aortas [76]. They found that VSMCs from AAA patients had impaired contraction compared to controls. This was consistent with Muratoglu et al. who reported that the VSMC-specific deletion of *Lrp1* (low-density lipoprotein receptor-related protein 1) led to spontaneous aortic aneurysms [77]. Subsequently, Au et al. discovered that LRP1 is responsible for maintaining the contractile phenotype in VSMCs by regulating calcium signaling events that protect against aortic aneurysm [78]. These studies suggest that loss of the contractile VSMC phenotype is an important phenomenon in AAA pathophysiology.

The relationship between chronobiological patterns and vascular disease has recently attracted research interest [79]. Traditionally, it was thought that neurons in the suprachiasmatic nucleus were solely responsible for circadian rhythmicity. However, it is now widely accepted that cells in the cardiovascular system are able to maintain their own circadian rhythmicity. Brain and muscle ARNT-like 1 (BMAL1) is a well-known transcription factor and obligatory clock gene that maintains circadian rhythmicity in peripheral tissues [80]. Interestingly, Lutshumba et al. found that VSMC-specific deletion of BMAL1 protected mice from aneurysm induction in two different models [81]. Further, they found that VSMC deletion of BMAL1 led to upregulated levels of tissue inhibitor of metalloproteinase 4 (TIMP4), which revealed an intriguing interaction between these two proteins in the pathogenesis of aortic aneurysms. VSMCs have also been shown to be a major contributor to the progression and remodeling of aortic dissections into aneurysms [82]. However, it has been challenging to identify VSMCs in diseased tissues due to phenotypic switching. Clément et al. used multicolor lineage tracing in *Myh11-CreERt2/Rosa26-Confetti* mice to track the fate of VSMCs upon induction of the Ang II model [83]. VSMCs were labeled via tamoxifen injections prior to aneurysm induction. They found that medial-VSMCs undergo clonal expansion and are primarily observed in the adventitia and borders of the false lumen in those that developed dissecting aneurysms. These VSMCs underwent phenotypic switching as evidenced by their upregulation of phagocytic markers, which correlated with increased severity of aortic disease. Deletion of autophagy protein 5 (*Atg5*) enhanced VSMC death and promoted inositol-requiring enzyme 1α-dependent inflammation, which was associated with increased severity of aortic disease. These results suggested that VSMC proliferation and autophagy promote aortic wall repair and limit the development of dissecting aortic aneurysms.

### 3.4 Endothelial cells

The endothelium is in direct contact with blood flow and acts as a barrier to the rest of the aortic wall. Although it was originally thought that the endothelial layer compromises homogenous population, Kalluri et. al utilized single-cell RNA sequencing to identify functionally distinct endothelial cell populations in mice [84]. Gene set enrichment analysis of the endothelial cells revealed a lymphatic cluster and two subpopulations that were more specialized in lipoprotein handling, angiogenesis, and extracellular matrix production, which persisted when mice were given a Western diet. Thus, this provided further insight into the complexity of the endothelial cell layer. Clinical studies have demonstrated that circulating biomarkers of endothelial dysfunction are closely related to the incidence of aortic aneurysms [85]. In addition, strong associations were found between the use of endothelial protective medications such as statins, angiotensin-converting enzyme inhibitors, and angiotensin receptor blockers and inhibition of aneurysm rupture or growth [86]. Along these lines, Rateri et al. discovered that attenuation of aortic aneurysms via AT1R is endothelial-specific. Mice lacking AT_1a_ receptor (AT1AR) in endothelial cells, not those lacking AT1AR in VSMCs, were protected from Ang II-induced aortic aneurysm [87]. Consistent with Rateri et al., Galatioto and colleagues found, via computational analyses of differentially expressed genes, that improper AT1AR activity in the vascular endothelium was a significant determinant of aneurysms in mice [88]. Therefore, angiotensin receptor blockers appear to be an excellent pharmaceutical candidate to repurpose for aortic aneurysm treatment.

## 4. Abutting Aortic Wall Entities

### 4.1 Perivascular adipose tissue

Other structural components outside of the vascular wall have been shown to have a significant impact on the pathogenesis of aortic aneurysms. Perivascular adipose tissue (PVAT) has been shown to have a role in vascular function and disease [89]. PVAT is in direct contact with the adventitia of the vessel and can regulate arterial homeostasis via autocrine/paracrine effects, tuning dilation and contractile functions, and regulating aortic wall inflammation [90]. Piacentini et al. compared the transcriptome of PVAT from the dilated portions of aortic tissue to the non-dilated portions in patients with aortic aneurysms [91]. The group found that the transcriptional landscape of PVAT of the dilated aorta was associated with dysfunctional immune/inflammatory response, which was highly suggestive of autoimmune mechanisms. This work highlighted the importance of PVAT in the pathogenesis of aortic aneurysms and suggested an immunomodulation strategy may be an effective treatment in these patients. However, it is still unclear whether the changes seen in the PVAT is a consequence or cause of aortic aneurysm development.

### 4.2 Intraluminal thrombus

While PVAT influences aortic aneurysm formation via its outer anatomic location, intraluminal thrombus (ILT) can affect aneurysm progression from its inner location. ILT was originally thought to be protective of rupture in aortic aneurysms by cushioning the effect of blood pressure on the aortic wall [92]. However, it is now well understood that the constituents of the ILT actually interact with the aortic wall and contribute to the pathophysiology of aortic aneurysms via secretion of biologically active molecules [93–94]. To further investigate the interaction between these two entities, Andersen et al. performed a proteomic analysis of human tissue samples collected from abdominal aortic aneurysms and thrombus at the time of operative repair [95]. The authors demonstrate that ECM proteins within the aorta and ILT were negatively associated with aneurysm growth rates. They found a positive correlation between growth rates and plasma proteins both within the ILT and aortic wall. This suggested that increased porosity of ILT may have led to plasma proteins diffusing into the aortic wall contributing to the pathology.

## 5. Conclusion

Maintenance of aortic health requires a delicate balance between the ECM, cellular components of the vascular wall, immune system, and abutting structures. Alterations in any of these entities initiates cascades of events that subsequently lead to the development of aortic aneurysms. Current management strategies rely on open or endovascular repair of these aneurysms. Thus, great potential exists for a pharmaceutical agent that may halt or hinder aneurysm progression. Although our understanding of this complicated disease process continues to grow, much has yet to be learned.
